# Notes on *Nilothauma* Kieffer from Oriental China, with descriptions of three new species (Diptera, Chironomidae)

**DOI:** 10.3897/zookeys.574.6129

**Published:** 2016-03-28

**Authors:** Xin Qi, Hongqu Tang, Xinhua Wang

**Affiliations:** 1 College of Life Science, Taizhou University, Taizhou, Zhejiang 318000, China; 2 Reasearch Center of Hydrobiology, Jinan University, Guangzhou 510632, China; 3 College of Life Science, Nankai University, Tianjin 300071, China

**Keywords:** Nilothauma, new species, new records, Oriental China, key

## Abstract

Three new species of *Nilothauma* Kieffer are described and figured from Oriental China: *Nilothauma angustum* **sp. n.** based on the male only, *Nilothauma aristatum* **sp. n.** based on the male, pupa and larva, and *Nilothauma bilobatum* **sp. n.** based on the male and pupa. In addition, new distribution records are given for *Nilothauma japonicum* Niitsuma, *Nilothauma nojirimaculatum* Sasa, *Nilothauma hibaratertium* Sasa, and *Nilothauma acre* Adam & Sæther. A key to known males of *Nilothauma* Kieffer in China is provided.

## Introduction

The genus *Nilothauma* Kieffer, 1921 is represented by 43 species: six species occurring in the Palaearctic region, four in the Nearctic region, 16 in the Neotropical region (not including *Nilothauma aleta* Roback and *Nilothauma duena* Roback due to the uncertain status), six species in the Oriental region, 11 species in the Afrotropical region, two species in the Australasian region, and two species occuring both in the Palaearctic and Oriental regions (Adam and Sæther 1999; Mendes and Andersen 2009; Qi et al. 2014). From China, five species have been recorded: *Nilothauma japonicum* Niitsuma, *Nilothauma nojirimaculatum* Sasa, *Nilothauma acre* Adam & Sæther, *Nilothauma quatuorlobum* Yan, Tang & Wang, and *Nilothauma pandum* Qi, Lin, Wang & Shao; all in the Oriental part of the country. No adult information is available on the genus from Palearctic parts of China.

In the present paper, we present new material of *Nilothauma* from Oriental China. Three species are described as new to China, and new distributional records are given for *Nilothauma acre* Adam & Sæther, *Nilothauma hibaratertium* Sasa, *Nilothauma japonicum* Niitsuma and *Nilothauma nojirimaculatum* Sasa. We also present an identification key to males of *Nilothauma* in China.

## Materials and methods

Descriptions of morphological characters are based on slide-mounted specimens in Euparal. Terminology for morphology and abbreviations follow Sæther (1980) and Adam and Sæther (1999).

Most of the specimens examined here are deposited in the College of Life Science, Taizhou University (LTZU) and partial in Nankai University (LNKU). The holotype specimens of three new species are deposited in the Ecology Department, Jinan University (EJNU).

## Taxonomy

### Nilothauma angustum sp. n.

Taxon classificationAnimaliaDipteraChironomidae

http://zoobank.org/6AEAD9D5-C373-4D0A-822A-5449C7A62C06

Figs 1–11

#### Type material.

Holotype: male (EJNU), CHINA: Yunnan, Ximeng City, Mengsuo Lake, 22°38.689'N, 99°35.631'E, Alt. 1090m, 27.viii.2014, Tang HQ, light trap. Paratype: 1 male (LTZU), as holotype.

#### Diagnosis.

The adult male of *Nilothauma angustum* sp. n. can be distinguished from all other known species of the genus by the following combination of characters: wing with four partially connected dark markings; anterior T IX projection extensively microtrichiose, divided into two lobes, each with apical simple setae forming a fan-like structure; posterior T IX projection extensively microtrichiose, nearly parallel-sided, setose, with long anterolateral arms; anal point broadly lanceolate, microtrichiose along the median ridge and the apical margin; median volsella with microtrichia and two apical setae; gonostylus peaked apically.

#### Etymology.

From the Latin *angustus* (narrow), referring to the male hypopygium with apically narrowed gonostylus.

#### Description.

Male imago (n = 2).

Total length 2.1−2.2 mm. Wing length 0.9−1.1 mm. Total length/wing length 2.1−2.2. Wing length/length of profemur 2.1−2.2.

**Figures 1–11.**
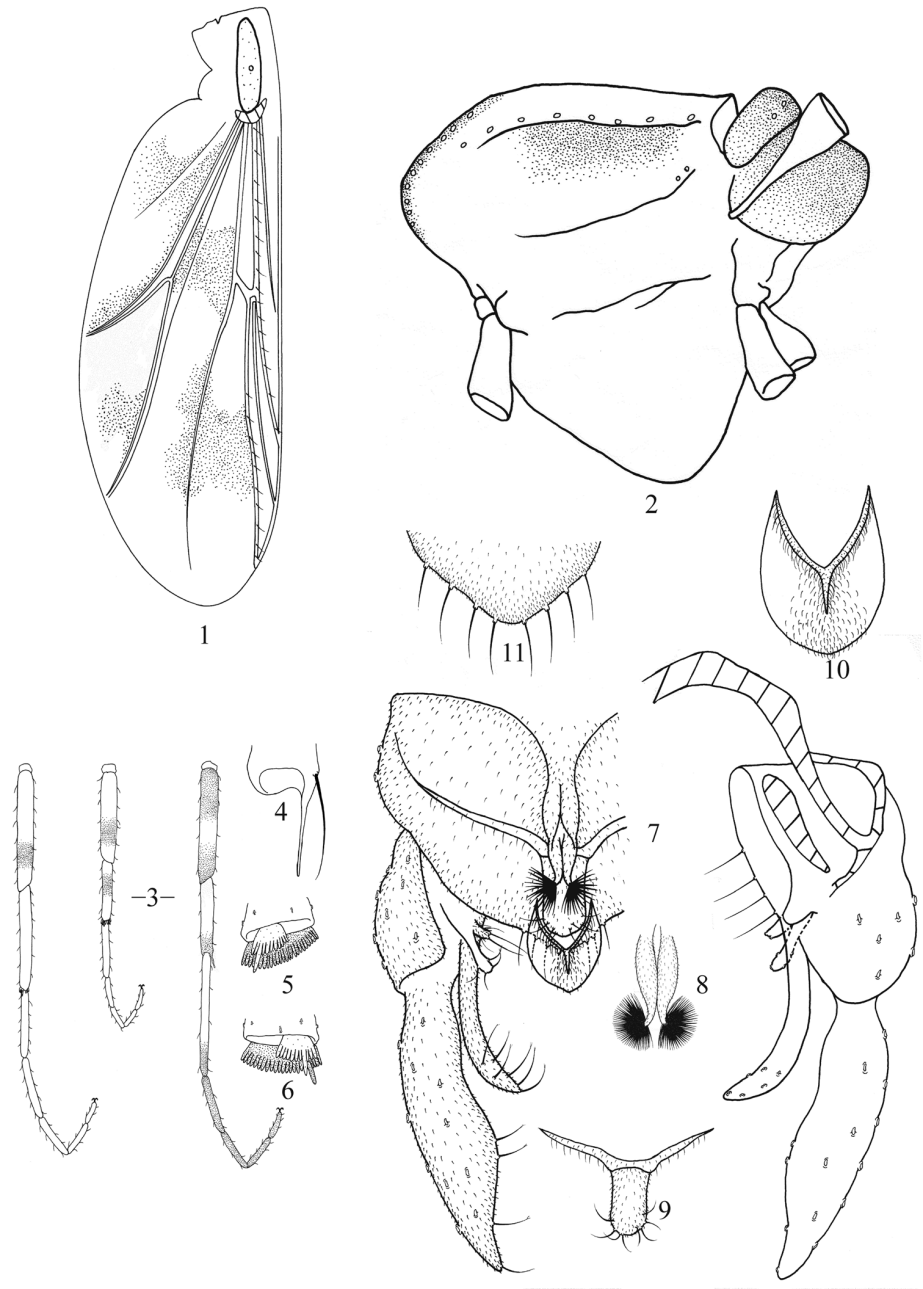
*Nilothauma angustum* sp. n., male. **1** wing **2** thorax, lateral view **3** legs **4** foretibial apex **5** mid tibial apex **6** hind tibial apex **7** hypopygium, dorsal view (left) and ventral view (right) **8** anterior anal tergal projection **9** posterior anal tergal projection **10** anal point **11** posterior margin of anal tergite.

*Coloration*. Generally yellow, thorax (Fig. 2) yellow except scutum, pre-episternum, scutellum and postnotum dark brown, abdomen yellowish brown. Wing with 4 partially connected dark markings (Fig. 1). Foreleg yellow with both ends of femur, apex of tibia, apical 1/3 of ta_1,_ and ta_2−5_ brown; mid leg with sub-apex of femur and sub-base of tibia brown; hind leg with sub-apex of femur brown (Fig. 3).

*Head*. AR 0.18−0.20. Temporals 7−11, uniserial. Clypeus with 17−19 setae. Tentorium 80−108 μm long, 11−15 μm wide; stipes 50−65 μm long, 6−8 μm wide. Lengths of palpomeres 1−5 (in μm): 28−30, 25−30, 58−63, 85−105, 125−130. Palpomere 3 with 2 sensilla clavata; Pm_5_/Pm_3_ 2.1−2.2.

*Thorax* (Fig. 2). Antepronotal lobe much reduced. Dorsocentrals 9−10, uniserial; acrostichals 7−9, biserial; prealars 2. Scutellum with 2 setae.

*Wing* (Fig. 1). VR 1.5−1.6. Brachiolum with 1 seta, R with 9−13 setae, R_1_ with 6−9 setae, R_4+5_ with 10−11 setae.

*Legs* (Fig. 3). Spur of fore tibia 63−65 μm long including 30−35 μm long scale (Fig. 4). Spur of mid tibia 20−25 μm long (Fig. 5) including 13−15 μm long comb. Spurs of hind tibia 18−25 μm and 28−30 μm long, respectively; comb 15−20 μm long (Fig. 6). Width at apex of fore tibia 28−30 μm, of mid tibia 33−35 μm, of hind tibia 30−34 μm. Lengths and proportions of legs in Table 1.

**Table 1.** Lengths (μm) and proportions of legs of *Nilothauma angustum* sp. n., male (n = 2).

	fe	ti	ta_1_	ta_2_	ta_3_	ta_4_	ta_5_	LR	BV	SV
P_1_	438−500	325−360	438−500	155−195	130−150	105−110	70−80	1.4	2.5−2.6	1.7
P_2_	450−500	325−338	185−190	80−100	60−75	50−63	50−60	0.56−0.58	3.5−4.4	3.6−4.1
P_3_	488−538	488−538	270−320	135−165	140−165	100−108	70−78	0.55−0.60	2.7−2.8	3.4−3.6

*Hypopygium* (Fig. 7). Tergite IX with 2 dorsal projections. Anterior projection (Fig. 8) microtrichiose, 60−63 μm long, 20−23 μm wide at base, split into 2 lobes; each 40−45 μm long, 10−12 μm wide at base, 5−6 μm wide at apex, with simple apical setae, together forming fan-like structure. Posterior projection (Fig. 9) extensively microtrichiose, 37−40 μm long, 35−40 μm wide at base, 17−20 μm wide at apex, nearly parallel-sided, apically rounded with 8 setae; long anterolateral arms present. Anal point (Fig. 10) broadly lanceolate, 30−35 μm long, 25−28 μm wide at base, 28−30 μm wide at middle, with microtrichia along median ridge and apical margin. Posterior margin of tergite IX (Fig. 11) with 8−10 setae located to each side of anal point. Laterosternite IX with 3 setae. Phallapodeme 28−30 μm long. Transverse sternapodeme rounded medially without median elongation. Gonocoxite 78−80 m long. Superior volsella 30−35 μm long, slender, club-shaped with 4 apical setae, without microtrichia. Median volsella 8−10 μm long, with 2 apical setae and microtrichia. Inferior volsella 53−58 μm long, pointed apically, with microtrichia and 6−7 apically cleft setae. Gonostylus 88−90 μm long, apically narrowed and peaked, with row of 4−5 split distal-median setae. HR 0.86−0.89, HV 2.3−2.5.

Female imago, pupa and larva. Unknown.

#### Remarks.

The male hypopygium is similar to those of *Nilothauma flabellatum* Adam & Sæther, 1999 and *Nilothauma kakumense* Adam & Sæther, 1999 as the anterior T IX projection has long apical setae forming fan-like structures. The differences between these three species are given in Table 2.

**Table 2.** Comparison of male hypopygial characters in *Nilothauma angustum* sp. n., *Nilothauma flabellatum* Adam & Sæther and *Nilothauma kakumense* Adam & Sæther.

	*Nilothauma angustum* sp. n.	*Nilothauma flabellatum*	*Nilothauma kakumense*
Anterior T IX projection	with simple setae	with apically branched setae	with apically widened and unbranched setae
Main part of posterior T IX projection	with microtrichia	without microtrichia	without microtrichia
Median volsella	with microtrichia	without microtrichia	without microtrichia
Anal point	without distal-median knob	with distal-median knob	with distal-median knob
Transverse sternapodeme	without median elongation	with median elongation	without median elongation

#### Distribution.

Oriental China (Yunnan Province).

#### Biological note.

The males were collected at the bank of Mengsuo Lake by light trap, where the nutrient levels are relatively high (conductivity 39−42 μs/cm, chlorophyll-a 10.5−11.1 μg/l). The co-occurring dominant species are eutrophic taxa, such as *Kiefferulus* sp., *Polypedilum nubeculosum* (Meigen), *Polypedilum sordens* (van der Wulp), and *Tanytarsus oscillans* Johannsen.

### Nilothauma aristatum sp. n.

Taxon classificationAnimaliaDipteraChironomidae

http://zoobank.org/53489B41-D9EC-4AA2-AC3B-B6001B819231

Figs 12–17 , 18–24

#### Type material.

Holotype: male with pupal exuviae (EJNU), CHINA: Anhui Province, Huangshan Nature Conservation Reserve, stream in Huang Mountain, 30°04.317'N, 118°09.320'E, Alt. 520 m, 4.v.2014, Tang HQ, light trap. Paratypes: 1 male (LTZU), CHINA: Zhejiang Province, Lin-An City, Tianmu Mountain, 16.vii.2012, Lin XL, hand net; male with larval and pupal exuviae (LTZU), reared by Lin XL, as previous; 3 pupal exuviae (EJNU), CHINA: Guangdong Province, Dongguan City, Yinping Nature Conservation Reserve, 22°53.772'N, 114°14.086'E, 17.iv.2012, Tang HQ, hand net.

#### Diagnosis.

The adult male of *Nilothauma aristatum* sp. n. can be distinguished from other known *Nilothauma* species by the anterior T IX projection with plumose setae; the anal point broadly lanceolate with microtrichia along the median ridge; the superior volsella slender with a lateral spur, and one lateral and 2−3 apical setae, without microtrichia. The pupa is characterized by the relatively short frontal setae (1.5−2.0 times as long as the major axis of basal ring); and the anal comb of abdominal segment VIII consisting of a main spur and a single accessory spine. The larva cannot be reliably separated from those of other species.

#### Etymology.

From Latin *aristatus* (aristate), referring to the male hypopygium with a lateral spur on the superior volsella.

#### Description.

Male imago (n = 2).

Total length 3.0−3.5 mm. Wing length 1.4−2.1 mm. Total length/wing length 1.7−2.2. Wing length/length of profemur 1.9−2.6.

**Figures 12–17.**
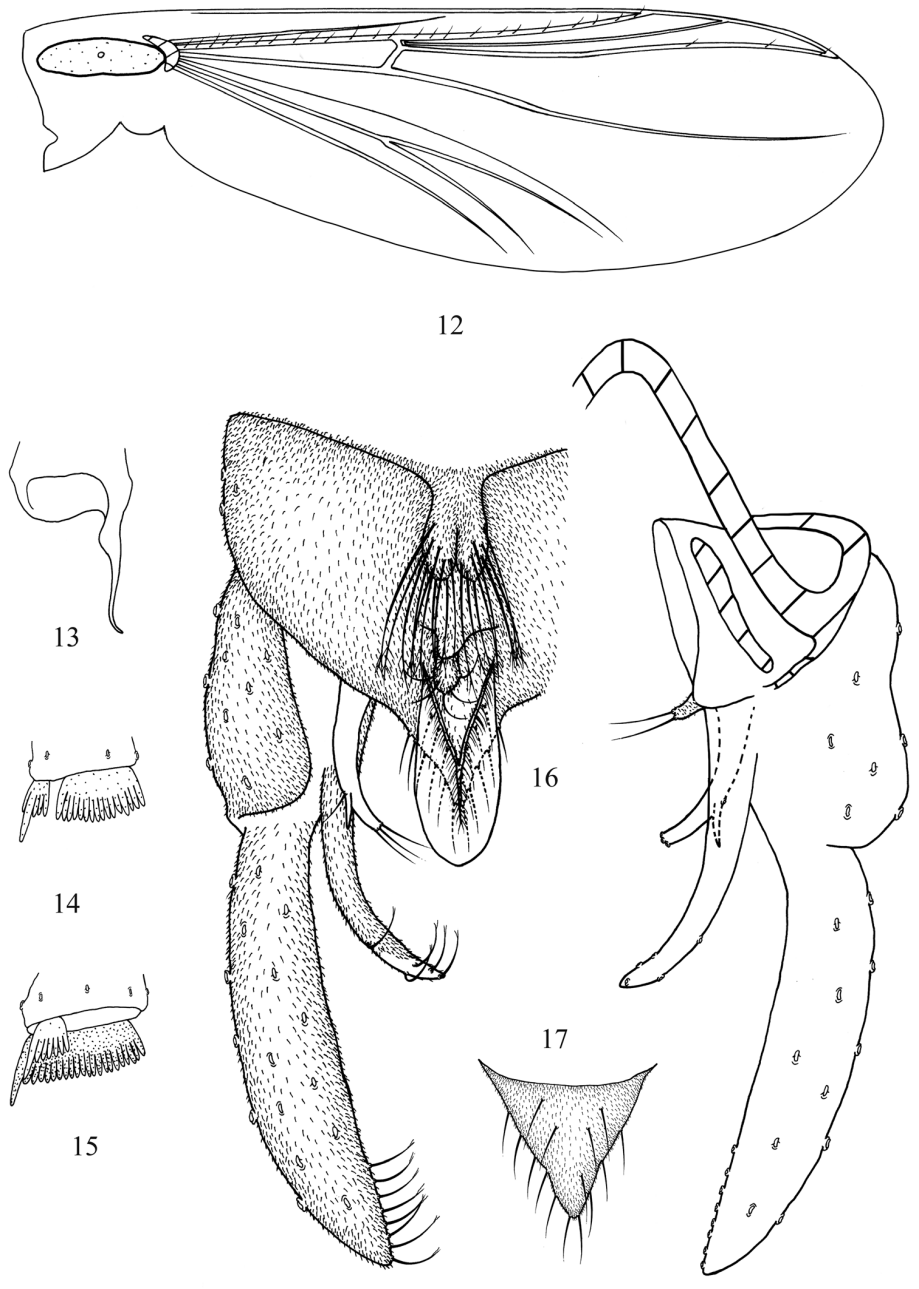
*Nilothauma aristatum* sp. n., male. **12** wing **13** foretibial apex **14** mid tibial apex **15** hind tibial apex **16** hypopygium, dorsal view (left) and ventral view (right) **17** posterior margin of anal tergite.

*Coloration*. Entirely pale yellow. Wing without any marking (Fig. 12).

*Head*. AR 0.16−0.21. Temporals 6. Clypeus with 10−13 setae. Tentorium 145−170 μm long, 21−23 μm wide. Stipes 85−90 μm long, 8−10 μm wide. Lengths of palpomeres 1−5 (μm): 30−32, 30−40, 70−80, 130−140, 155−160. Palpomere 3 with 2 sensilla clavata, longest 10 μm long. Pm_5_/Pm_3_ 1.9−2.3.

*Thorax*. Dorsocentrals 5−7, acrostichals 10−15, prealars 2−3, scutellars 1−2.

*Wing*. VR 1.4. Brachiolum with 1 seta, R with 13−15 setae, R_1_ with 11 setae, R_4+5_ with 3−4 setae.

*Legs*. Spur of foretibia 68−75 μm long including 30−43 μm long scale (Fig. 13). Spur of mid tibia 30−38 μm long (Fig. 14) including 16−25 μm long comb. Spurs of hind tibia 30−38 μm and 37−40 μm long, respectively (Fig. 15); comb 15−28 μm long. Width at apex of foretibia 40−50 μm, of mid tibia 45−50 μm, of hind tibia 43−54 μm. Lengths and proportions of legs in Table 3.

**Table 3.** Lengths (μm) and proportions of legs of *Nilothauma aristatum* sp. n., male (n = 2).

	fe	ti	ta_1_	ta_2_	ta_3_	ta_4_	ta_5_	LR	BV	SV
P_1_	725−788	538−575	725−775	375−450	310−375	260−288	125−150	1.4	1.7−1.9	1.7−1.8
P_2_	625	450−525	275−350	140−150	100−125	70−75	60−75	0.61−0.67	3.5−3.7	3.3−3.9
P_3_	750−800	725−800	375−488	200−250	200−250	150−163	85−88	0.52−0.61	2.8−2.9	3.3−3.9

*Hypopygium* (Fig. 16). Tergite IX with 2 dorsal projections. Anterior projection completely divided into 2 oval lobes; each 35−37 μm long, 12−13 μm wide at middle, with 8−10 plumose setae 50−63 μm long. Posterior projection 10−12 μm long, 10−13 μm wide at base, 5−6 μm wide at apex, apically rounded, with 5 setae 13−20 μm long. Anal point very broadly lanceolate, 50−60 μm long, 18−20 μm at base, 25−27 μm at middle, with microtrichia along median ridge. Posterior margin of tergite IX (Fig. 17) with 9−11 setae. Laterosternite IX with 3 setae. Phallapodeme 37−40 μm long. Transverse sternapodeme without median elongation. Gonocoxite 114−120 μm long. Superior volsella 45−50 μm long, with lateral spur, and one lateral and 2−3 apical setae, without microtrichia. Median volsella 10−13 μm long, bearing 2 apical setae and microtrichia. Inferior volsella 78−90 μm long, curved dorsally, pointed apically, with microtrichia and 5 apically branched setae. Gonostylus 110−130 μm long, with 8 split median setae in distal 1/3. HR 1.02−0.92, HV 2.69−2.73.

Pupa (n = 4).

Total length 3.5−4.4 mm. Exuviae pale brown with anal comb on abdominal segment VIII yellowish brown.

*Cephalothorax*. Frontal seta short, 30−50 μm long (n = 2). Basal ring small, stoma-like, with major axis 20−25 μm long, minor axis 5−8 μm high. Frontal setae 1.8−2.0 times as long as major axis of basal ring. Thorax pebbled and rugose dorsally.

**Figures 18–24.**
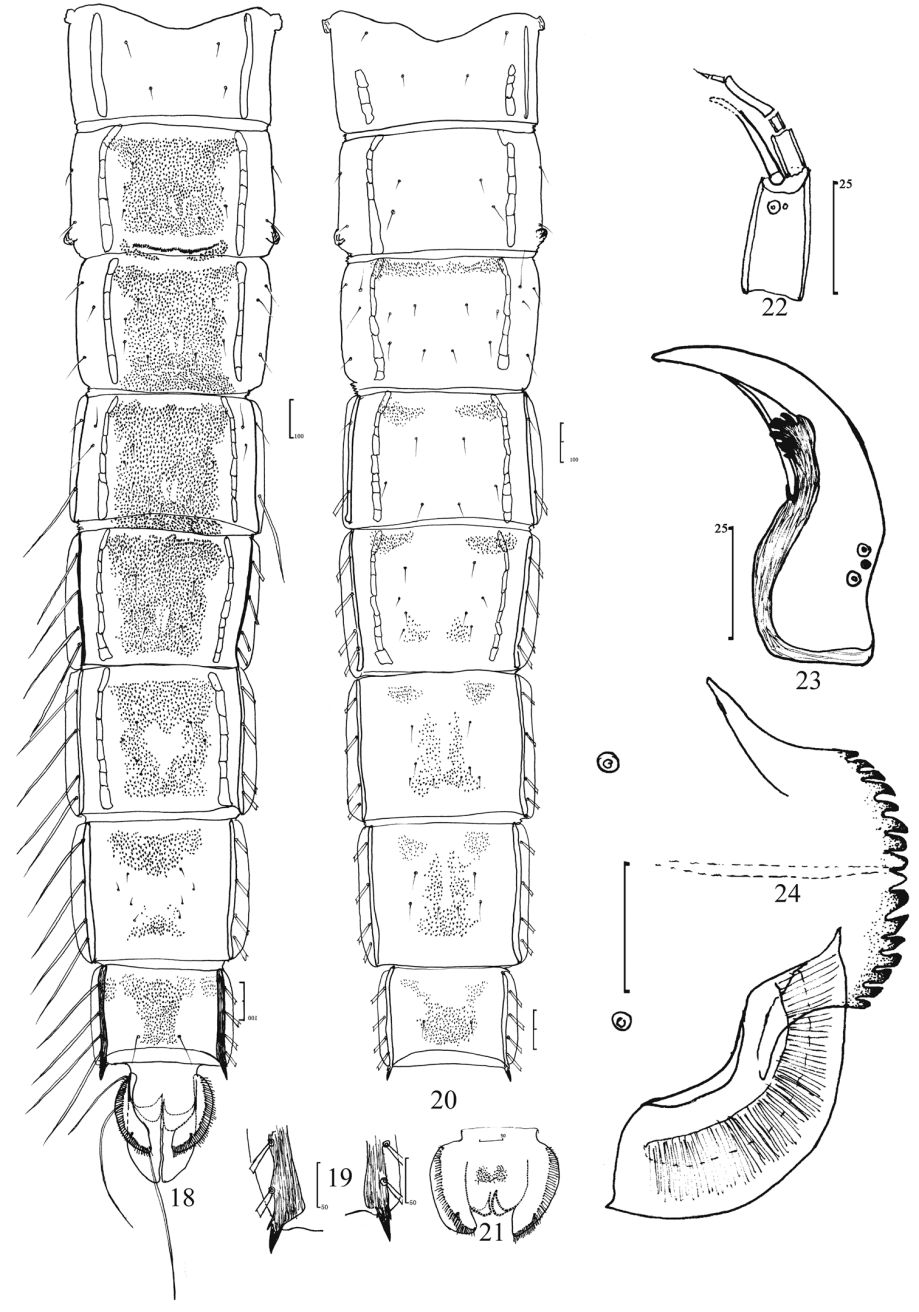
*Nilothauma aristatum* sp. n., pupa (**18–21**) and larva (**22–24**). **18** dorsal view of the abdomen (male) **19** anal comb of abdominal segment VIII, showing combs of both sides **20** ventral view of abdomen I–VIII **21** dorsal view of abdomen IX (female) **22** antenna **23** mandible **24** mentum.

*Abdomen* (Fig. 18). Tergite I without spinulation; T II−VI extensively spinulated; T VII with anterior and posterior bands of spines; T VIII with anterolateral and median bands of spines; tergite T IX with median spinulation in female (Fig. 21), but without any spinulation in male. S I−II without spinulation; S III−IV with anterior spinulation; sternite IV with weak anterolateral spinulation; S V with weak anterolateral and caudolateral spinulation; S VI−VIII with anterolateral and median spinulation, occasionally anterolateral spinulation merged to median in S VIII (Fig. 20). T II with row of 70−78 caudal hooklets with posterior groups of points behind each end. Conjunctives III/IV and IV/V with rows of spinules. Pedes spurii B weakly developed on segment II. Anal comb of segment VIII (Fig. 19) composed of main spur 20−30 μm long and single accessory spine 7.5−17.5 μm long. Segment I without L-setae; segments II–III each with 3 L-setae on each side; segment IV with 2 L-setae and 1 LS-seta on each side; segments V–VIII each with 4 LS-setae on each side. Anal lobe 200−240 μm long, 2.4−2.6 times as long as broad, with 35−48 lateral setae, dorsal seta located near distal 1/3.

Larva (n = 1).

Total length 5 mm. Head capsule about 300 μm long, about 260 μm wide.

*Coloration*. Red color in fresh specimens, head pale yellow. Mentum and postoccipital margin brown.

*Antenna* (Fig. 22). Lengths of antennal segments 1–6 (μm): 28, 10, 4, 13, 4, 4. AR 0.8. Basal segment with ring organ situated in distal 1/6; antennal blade 25 μm long, extending to apex of segment 4; segment 6 hair-like, almost as long as segment 5.

*Mandible* (Fig. 23). Total length 85 μm. Apical tooth 40 μm long; 4 inner teeth small, arising from common base. Seta subdentalis 30 μm long, reaching middle of apical tooth.

*Mentum* (Fig. 24). Width 55 μm. Two pale median teeth and 7 pairs of gradually decreasing lateral teeth present. Ventromental plate 65 μm wide.

Female imago. Unknown.

#### Remarks.

The male is similar to that of Oriental species *Nilothauma acre* Adam & Sæther, 1999 in having the wing unmarked, the anterior T IX projection with plumose setae, the anal point lanceolate, and the superior volsella slender with a lateral spur and one lateral and two three apical setae. It differs from it as the anal point bears microtrichia along the median ridge, the superior volsella is relatively long compared to the median volsella (length ratio, Svo/Mvo > 4.0) and the inferior volsella has simple setae only. In *Nilothauma acre*, the anal point is bare, length of Svo/Mvo is around 2.0 and the inferior volsella has apically split setae.

The pupa of *Nilothauma aristatum* sp. n. will key to “*Nilothauma* sp. Australia” in Adam and Sæther (1999), but may be separable by the relatively short frontal setae. The ratio of the length of the frontal seta to the length of the major axis of basal ring is 1.8−2.0 in *Nilothauma aristatum* sp. n., but 4.6−6.5 in the latter. The larva of *Nilothauma aristatum* sp. n. somewhat resembles that of *Nilothauma japonicum* Niitsuma, 1985, but it remains uncertain because of a paucity of data.

#### Distribution.

Oriental China (Anhui, Guangdong and Zhejiang Provinces).

#### Biological note.

The larva and pupa of *Nilothauma aristatum* sp. n. are found in first-, or second-order streams. The water is relatively clean and cold (water temperature 15°C−20°C, pH 7.80−7.88, DO% 90.6−93.4, DO 8.09−9.36 mg/l, and conductivity 25−34 μs/cm). The co-existing dominant species of chironomids are *Eukiefferiella* spp., *Rheotanytarsus* spp., *Rheocricotopus* spp., and *Parametriocnemus* spp. Some steno-thermic species, such as *Heleniella* sp. and *Pagastia* sp., are frequently observed in the pupal exuviae samples.

### Nilothauma bilobatum sp. n.

Taxon classificationAnimaliaDipteraChironomidae

http://zoobank.org/191CECE5-B1B0-4BE0-A649-67F7EAB2B4CE

Figs 25–32 , 33–40

#### Type material.

Holotype: male with associated pupal exuviae (EJNU), CHINA: Guangxi Zhuang Autonomous Region, Guilin City, Qingshitan Reservoir, 25°31.640'N, 110°13.499'E, Alt. 235 m, 26.viii.2014, Long Term Ecology Research Group (LTER), light trap. Paratypes: 2 males with pupal exuviae as holotype (EJNU); 1 male and 1 female pupa (EJNU), CHINA: Guangdong Province, Shantou City, Nan’ao county, Shen-Ao Reservoir, 23°28.390'N, 117°06.683'E, Alt. 61m, 17.iv.2015, Tang HQ, light trap.

#### Diagnosis.

The male of *Nilothauma bilobatum* sp. n. can be distinguished from other *Nilothauma* species by the following combination of characters: anterior T IX projection bearing simple setae only; anal point broadly lanceolate with microtrichia; superior volsella with a lateral spur, a main lobe bearing 4−5 apical setae, and a blunt-tipped lobe bearing a terminal seta, without microtrichia. The pupa can be separated from others by the following characters: relatively short frontal setae (as long as or slightly longer than the major axis of basal ring); and anal comb of abdominal segment VIII consisting of a main spur and 2−3 accessory spines.

#### Etymology.

From Latin *bi*- (two) and *lobatus* (lobate), referring to the male hypopygium with two lobes in the superior volsella.

#### Description.

Male imago (n = 4).

Total length 2.4−3.1 mm. Wing length 1.2−1.6 mm. Total length/wing length 1.6−2.7. Wing length/length of profemur 2.0−2.5.

*Coloration*. Generally pale yellow. Wing without any marking. Foreleg entirely yellowish brown; mid and hind legs with femora and tibiae pale yellow, and tarsus yellowish brown.

*Head*. AR 0.18−0.19. Temporals 7−10. Clypeus with 12−13 setae. Tentorium 100−125 μm long, 15−25 μm wide. Stipes 120−130 μm long, 5−8 μm wide. Lengths of palpomeres 1−5 (μm): 18−25, 33−37, 55−65, 100−125, 123−165. Palpomere 3 with 2 sensilla clavata; Pm_5_/Pm_3_ 2.2−2.5.

*Thorax*. Dorsocentrals 9−11, acrostichals 6−10, prealars 2−3, scutellars 2.

**Figures 25–32.**
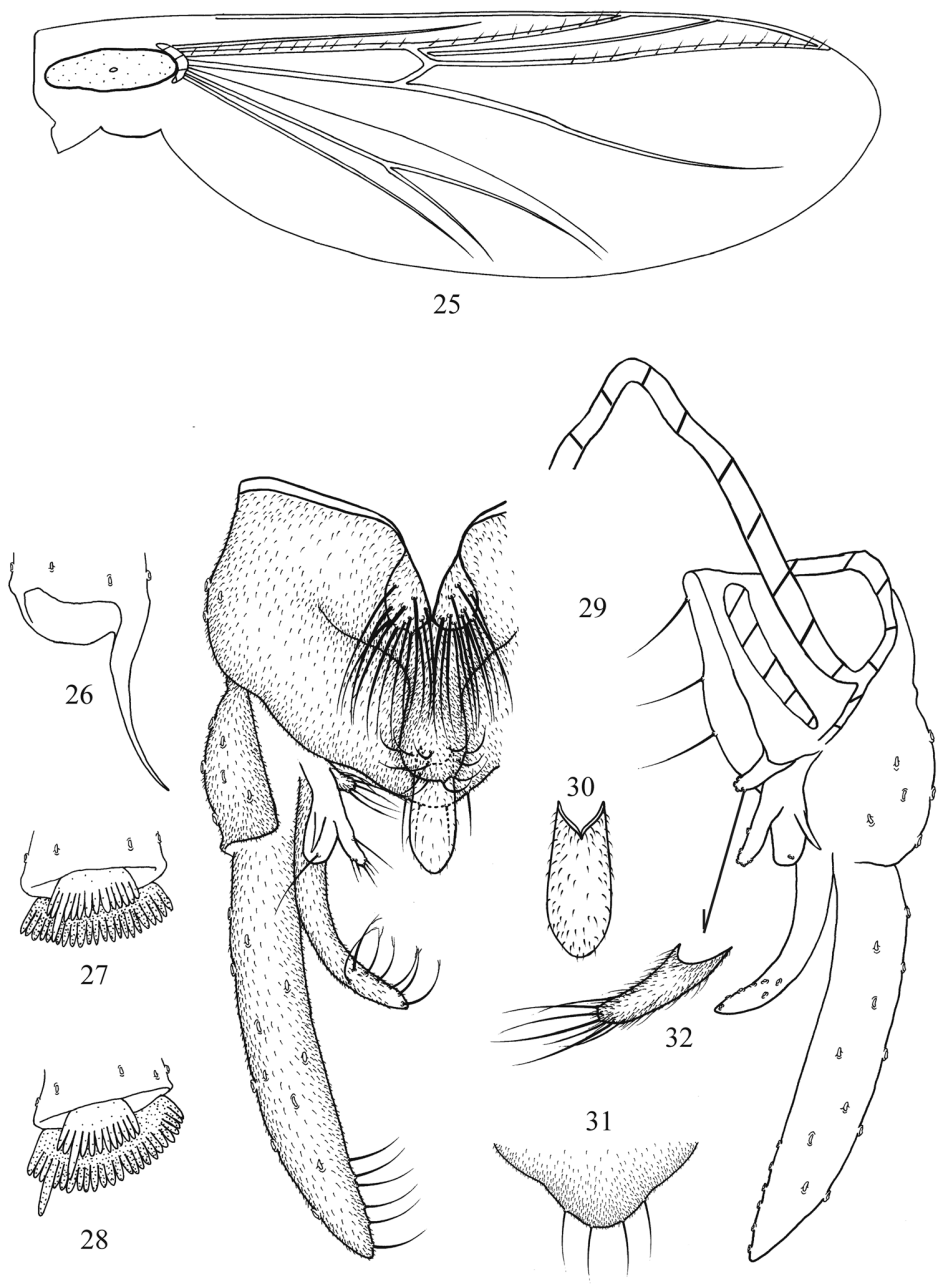
*Nilothauma bilobatum* sp. n., male. **25** wing **26** foretibial apex **27** mid tibial apex **28** hind tibial apex **29** hypopygium, dorsal view (left) and ventral view (right) **30** anal point **31** posterior margin of anal tergite **32** median volsella.

*Wing* (Fig. 25). VR 1.3−1.6. Brachiolum with 1 seta, R with 11−13 setae, R_1_ with 8−11 setae, R_4+5_ with 13−17 setae.

*Legs*. Spur of foretibia 60−80 μm long including 28−38 μm long scale (Fig. 26). Spur of mid tibia 22−25 μm long including 15−23 μm long (Fig. 27). Spurs of hind tibia 27−35 μm and 33−47 μm long, respectively (Fig. 28); comb 17−24 μm long. Width at apex of foretibia 34−42 m, of mid tibia 41−52 m, of hind tibia 48−50 m. Lengths and proportions of legs in Table 4.

**Table 4.** Lengths (μm) and proportions of legs of *Nilothauma bilobatum* sp. n., male (n = 4).

	fe	ti	ta_1_	ta_2_	ta_3_	ta_4_	ta_5_	LR	BV	SV
P_1_	625−650	475−513	663−700	300−360	280−300	210−240	120−130	1.3−1.4	1.8−1.9	1.6−1.9
P_2_	530−650	390−475	230−290	90−130	83−100	48−70	45−70	0.59−0.66	3.8−4.2	3.7−4.0
P_3_	600−725	600−700	290−400	180−210	175−210	120−150	75−100	0.48−0.60	2.7−2.8	3.3−4.1

*Hypopygium* (Fig. 29). Tergite IX with 2 dorsal projections. Anterior projection completely divided into 2 oval lobes; each 35−55 μm long, 8−10 μm wide at middle, with 12−15 simple setae 30−50 μm long. Posterior projection 28−32 μm long, 50−65 μm wide at base, 8−15 μm wide at apex, apically rounded, with 11−13 setae 20−25 μm long. Anal point (Fig. 30) very broadly lanceolate, 35−50 μm long, 13−20 μm at base, 15−20 μm at middle, with microtrichia. Posterior margin of tergite IX (Fig. 31) with 4−6 setae. Laterosternite IX with 3 setae. Phallapodeme 38−50 μm long. Transverse sternapodeme medially triangular, but without median elongation. Gonocoxite 100−120 μm long. Superior volsella 30−38 μm long, trifid; with lateral spur, main lobe bearing 4−5 apical setae, and blunt-tipped lobe terminating in seta; without microtrichia. Median volsella (Fig. 32) 20−30 μm long, with microtrichia and 4−6 apical setae. Inferior volsella 80−94 μm long, pointed apically, microtrichiose, with 7−8 simple apically split setae. Gonostylus 130−160 μm long, with 7−10 simple median setae in distal 1/3. HR 0.63−0.88, HV 1.5−2.4.

Pupa (n = 4).

Total length 5.0−5.6 mm. Exuviae yellow with posterior antepronotum and anal comb on abdominal segment VIII brown.

**Figures 33–40.**
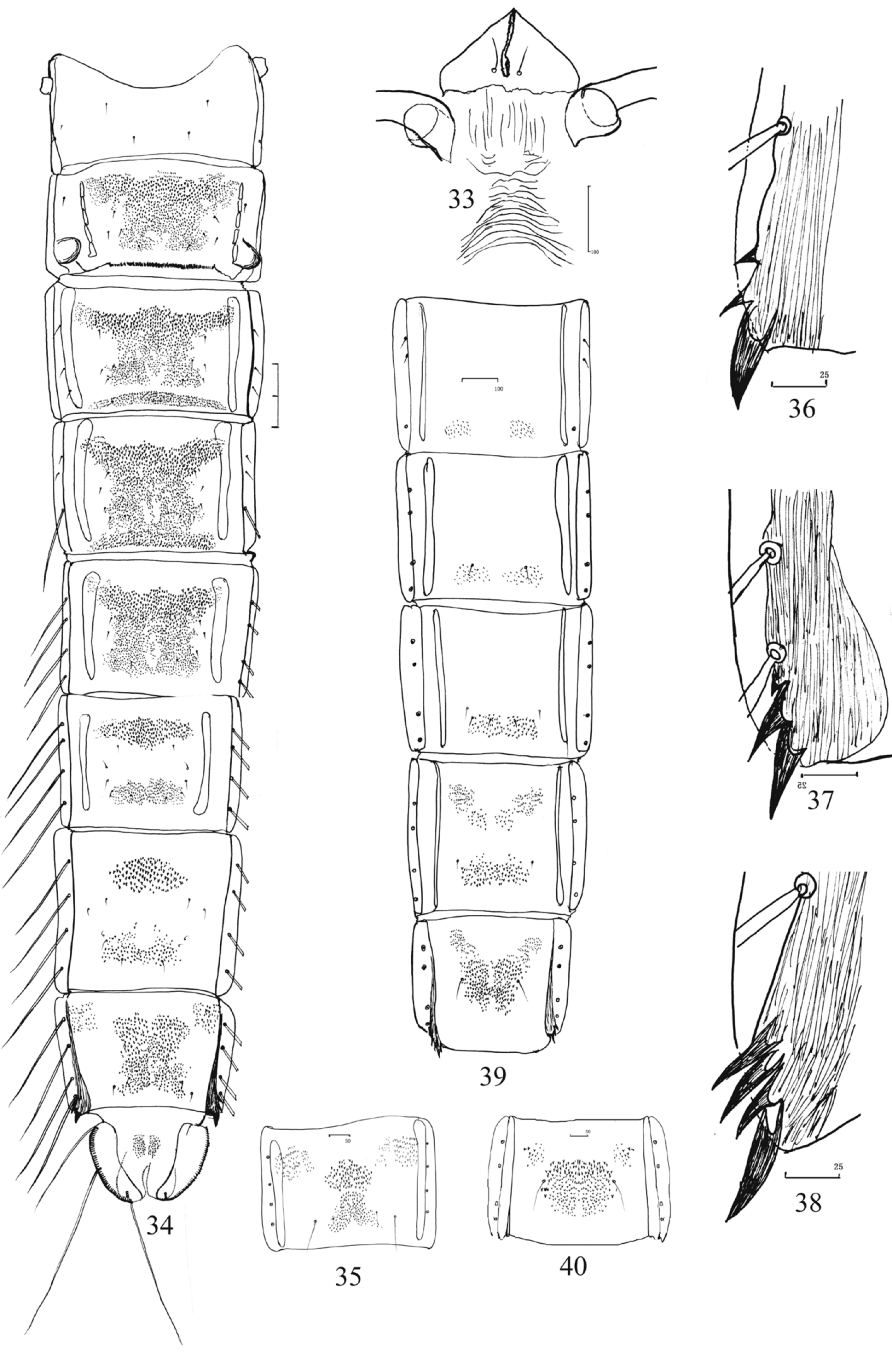
*Nilothauma bilobatum* sp. n. pupa. **33** frontal apotome **34** female abdomen, dorsal view **35** abdominal segment VIII, showing variation of tergal spinulation **36–38** anal comb of abdominal segemnt VIII, showing variation **39** the ventral view of abdomen IV–VIII **40** the ventral view of abdomen VIII, showing variation of sternal spinulation.

*Cephalothorax* (Fig. 33). Frontal apotome smooth. Frontal seta short, 38−40 μm long (n = 2). Basal ring oval with major axis 30−40 μm long, the posterior usually with 2−3 small tubercles. Frontal seta 1.0−1.2 times as long as major axis of basal ring. Thorax with one patch of small granules on each side of median suture.

*Abdomen* (Fig. 34–35). T I without spinulation; T II−V extensively spinulated; T VI−VII with anterior and posterior bands of spinules; T VIII with anterolateral and median spinulation; T IX with median spinulation in female pupa (Fig. 34), but without any spinulation in male. Anterior spinulation on T II–VIII consisting of somewhat large spinules. S I−III and IX without spinulation; S IV−VI with weak posterolateral spinulation; S VII−VIII with weak anterolateral and strong median spinulation, occasionally these merging into extensive spinulation in S VIII (Fig. 39, 40). Tergite II with row of 60−85 caudal hooklets. Conjunctives III/IV and IV/V with rows of spinules. Pedes spurii B distinct on segment II. Anal comb of segment VIII (Fig. 36–38) composed of main spur 30−50 μm long, and 2 or 3 accessory spines 10−30 μm long. Anal lobe 250−280 μm long, 1.8−2.2 times as long as broad, with 41−50 lateral setae, dorsal setae located near the distal margin of disc.

Female imago and larva. Unknown.

#### Remarks.

The male of *Nilothauma bilobatum* sp. n. is similar to that of *Nilothauma mirabile* (Townes, 1945) as the superior volsella has a lateral spur and two setigerous lobes, but separable by the anterior T IX projection bearing simple setae only and the anal point covered with microtrichia. In *Nilothauma mirabile*, the anterior projection has apically plumose setae and the anal point is bare. The pupa of *Nilothauma bilobatum* sp. n., as well as that of *Nilothauma aristatum* sp. n., will key to “*Nilothauma* sp. Australia” in Adam and Sæther (1999). The pupa resembles that of *Nilothauma aristatum* sp. n., rather than that of *Nilothauma* sp. Australia, in having relatively short frontal setae (1.0−1.2 times as long as the major axis of basal ring), but differs in the anal comb of abdominal segment VIII consisting of a main spur and 2−3 accessory spines. In *Nilothauma aristatum* sp. n., the anal comb has a main spur and a single accessory spine.

#### Distribution.

Oriental China (Guangxi Zhuang Autonomous Region and Guangdong Province).

#### Biological note.

The material was collected from two relatively eutrophic reservoirs (conductivity 24−65 μS/cm, dissolved oxygen 6.6−8.3 mg/l). The adults of the following species also occurred from there: *Glyptotendipes tokunagai* Sasa, *Dicrotendipes pelochloris* (Kieffer), *Tanytarsus oscillans* Johannsen, *Cladotanytarsus paratridorsus* Wang & Guo, and *Polypedilum masudai* (Tokunaga).

### Nilothauma acre

Taxon classificationAnimaliaDipteraChironomidae

Adam & Sæther

Nilothauma acre Adam & Sæther, 1999: 69.

#### Material examined.

2 males (LNKU), Jiangxi Province, Qianshan County, 13.vi.2004, Yan CC, light trap; 4 males (LTZU), Zhejiang Province, Taishun County, Wuyanling Natural Conservation Reserve, 1.viii.2005, Qi X, light trap; 1 male (LTZU), Zhejiang Province, Lin-An City, Tianmu Mountain, 16.vii.2012, Lin XL, sweep net.

#### Remarks.

This species was described from Fujian Province in China for the first time by Adam and Sæther (1999).

#### Distribution.

Oriental China (Fujian, Jiangxi, and Zhejiang Provinces).

### Nilothauma hibaratertium

Taxon classificationAnimaliaDipteraChironomidae

Sasa

Nilothauma hibaratertia Sasa, 1993: 73.
Tosayusurika simantofea Sasa, Suzuki & Sakai, 1998: 52
Nilothauma hibaratertium Sasa: Adam and Sæther 1999: 71.

#### Material examined.

1 male (EJNU), Yunnan Province, Mengla County, Menglun Town, Luosuo River at Xishuang Banna Tropical Botanical Garden, 29.viii.2014, Tang HQ, light trap; 2 males (EJNU), Anhui Province, Huangshan Nature Conservation Reserve, Fuxi stream, 25.v.2012, Tang HQ, light trap; 1 male (EJNU), Guangdong Province, Jiangmen City, Beifengshan Nature Conservation Reserve, 7.vii.2012, Tang HQ, light trap; 2 males (LTZU), Zhejiang Province, Jiangshan City, 12.viii.2012, Lin XL, sweep net; 1 male (LTZU), Zhejiang Province, Linan City, Tianmu Mountain, 16.vii.2012, Lin XL, sweep net; 1 male (EJNU), Fujian Province, Longqishan Nature Conservation Reserve, 14.xi.2012, Tang HQ, light trap; 2 males (EJNU), Fujian Province, Meihuashan Nature Conservation Reserve, 16.xi.2012, Tang HQ, light trap; 1 male (EJNU), Hainan Province, Bawangling Nature Conservation Reserve, 30.iv.2012, Tang HQ, light trap.

#### Remarks.

*Nilothauma hibaratertium* has never been described sufficiently, especially in the coloration of the adult. Examination of fresh specimens showed that the foreleg of the adult has distinct dark markings on the base and sub-apex of femora, and the apices of tibia and tarsomere 1. This is the first record of *Nilothauma hibaratertium* from the Oriental region; previously, this species has only been recorded from Palaearctic Japan (Yamamoto and Yamamoto 2014).

#### Distribution.

Oriental China (Yunnan, Anhui, Guangdong, Zhejiang, Fujian, and Hainan Provinces); Palaearctic Japan.

### Nilothauma japonicum

Taxon classificationAnimaliaDipteraChironomidae

Niitsuma

Nilothauma japonicum Niitsuma, 1985: 230.
Kribioxenus jintuprimus Sasa, 1990: 32.
Nilothauma jintuprima (Sasa): Sasa and Kikuchi, 1995: 34.

#### Material examined.

1 male (LTZU), Zhejiang Province, Linhai City, Sanjiang wetland,01.VI.2010, Li YF, sweep; 1 male (EJNU), Hainan Province, Jianfengling Nature Conservation Reserve, 29.iv.2012, Tang HQ, sweep net.

#### Remarks.

So far this species has been recorded from Thailand, Zhejiang and Hainan Province in China, as well as Palaearctic Japan (Adam and Sæther 1999; Yan et. al. 2005; Yamamoto and Yamamoto 2014).

#### Distribution.

Oriental China (Zhejiang, Hainan province); Thailand; Palaearctic Japan.

### Nilothauma nojirimaculatum

Taxon classificationAnimaliaDipteraChironomidae

Sasa

Nilothauma nojirimaculatum Sasa, 1991: 82.

#### Material examined.

1 male (EJNU), Hainan Province, Diaoluoshan Natural Conservation Reserve, 27.iv.2012, Tang HQ, light trap; 1 male (EJNU), Guangdong Province, Conghua City, Yugongdong Reservoir, 19.iii.2014, Tang HQ, light trap; 1 male (EJNU), Guangdong Province, Conghua City, Dongkeng Reservoir, 18.x.2014, Tang HQ, light trap.

#### Remarks.

This species was described from Palaearctic Japan and later recorded from Hainan in China (Adam and Sæther 1999).

#### Distribution.

Oriental China (Hainan and Guangdong Provinces); Palaearctic Japan.

### Key to males of the genus *Nilothauma* Kieffer in China

**Table:** 

1	T IX with one dorsal projection	***Nilothauma japonicum* Niitsuma**
–	T IX with two dorsal projections	**2**
2	Wing with dark markings	**3**
–	Wing without any marking	**4**
3	Anterior T IX projection with microtrichia	***Nilothauma angustum* sp. n.**
–	Anterior T IX projection without microtrichia	***Nilothauma nojirimaculatum* Sasa**
4	Superior volsella with one lateral spur or spinose branch	**5**
–	Superior volsella without spur or spinose branch	**8**
5	Anterior T IX projection undivided	***Nilothauma quatuorlobum* Yan, Wang & Tang**
–	Anterior T IX projection divided into two lobes	**6**
6	Anal point without microtrichia	***Nilothauma acre* Adam & Sæther**
–	Anal point with microtrichia	**7**
7	Superior volsella with two lobes and one lateral spur	***Nilothauma bilobatum* sp. n.**
–	Superior volsella with one lateral spur, without lobes	***Nilothauma aristatum* sp. n.**
8	Anal point with microtrichia	***Nilothauma pandum* Qi, Lin, Wang & Shao**
–	Anal point without microtrichia	***Nilothauma hibaratertium* Sasa**

## Supplementary Material

XML Treatment for Nilothauma angustum

XML Treatment for Nilothauma aristatum

XML Treatment for Nilothauma bilobatum

XML Treatment for Nilothauma acre

XML Treatment for Nilothauma hibaratertium

XML Treatment for Nilothauma japonicum

XML Treatment for Nilothauma nojirimaculatum
